# Effect of Tomato, Beetroot and Carrot Juice Addition on Physicochemical, Antioxidant and Texture Properties of Wheat Bread

**DOI:** 10.3390/antiox11112178

**Published:** 2022-11-03

**Authors:** Marianna Raczyk, Bartosz Kruszewski, Ewa Zachariasz

**Affiliations:** 1 Institute of Animal Reproduction and Food Research, Polish Academy of Sciences, 10-748 Olsztyn, Poland; 2 Department of Food Technology and Assessment, Institute of Food Sciences, Warsaw University of Life Sciences-SGGW, Nowoursynowska 159 C, 02-776 Warsaw, Poland

**Keywords:** wheat bread, tomato, carrot, beetroot, polyphenols, color, texture, sensory evaluation

## Abstract

Bakery products, including bread, are important components of the diet of people all over the world. One of the food industry’s goals is to improve its quality in the context of healthiness and physical parameters. Consumers’ perception of sensory quality is an important aspect of food choice. Thus, the study aimed to enhance nutritional parameters (antioxidant capacity, content of phenolic compounds) together with maintaining or increasing bread quality (texture, color, volume and sensory properties). Among vegetable juices, tomato, beetroot and carrot were selected, as they are easily accessible in Europe and are inexpensive. At the same time, those juices are known to be high in antioxidants. In this study, the effect of substituting recipe water with tomato, beetroot and carrot juices (replacement level: 15, 30, 50% *v*/*v*) was evaluated in terms of the specific volume, texture, color, acidity, polyphenol contents, antioxidant and sensory properties. It was concluded that juice content had a significant positive impact on physicochemical parameters such as volume, color, acidity, as well as the antioxidant activity of breads. The carrot and beetroot juices were the most efficient in terms of shaping wheat bread properties, especially in terms of antioxidant activity.

## 1. Introduction

According to the World Health Organization (WHO) recommendations, a healthy diet should be based on vegetables and whole grain products, among other things [1]. Keeping in mind that wheat (*Tritium aestivum* L.) bread is a staple food consumed all over the world, we decided to include vegetable juices in the bread making process and evaluate their effect on product quality. Bread may be a good carrier of bioactive compounds as it is a highly accepted, convenient food. However, vegetables added to baked products significantly change the physical and chemical characteristics [2,3] of it. In this study, a different amount of vegetable juices were evaluated to find out the best ratio which allows to keep consumers’ acceptance and quality parameters. In that way, in addition to the nutritional value increase, the organoleptic characteristics and overall quality of the product were expected to improve.

Consumer requirements of food quality play an important role in sensory perception and the determination of food acceptability. Bread acceptability is affected by texture, color, flavor, volume, shape, freshness, availability and price. Based on the literature, the health and sensory properties of a food product significantly influence consumer choice [4]. The usage of natural ingredients as a source of antioxidants and functional additives in bread and other bakery products is a global trend [5]. In bread and other baking goods production mostly white, cleaned flour is used; however, phenolic compounds are mostly (above 80% of total content) present in bran and germ fractions [6]. In wheat, phenolic compounds exist in soluble-free, soluble-conjugated, and insoluble-bound forms [7].

Vegetable juices compared to fresh products are available regardless of the season and have a longer shelf life. However, because of technological processes, juices have lower nutritional value compared to fresh vegetables. Conventional juice production with the mechanical pressing of the mash results in a slightly cloudy juice and a pomace.

Among vegetable juices, carrots and tomatoes are mostly selected by consumers worldwide [8,9]. Vegetable juices are a good source of flavonoids, plant pigments, vitamins and minerals, and in the case of natural unclarified juices, also dietary fiber. They have antioxidant properties and can have a positive effect on the condition of the cardiovascular system, which reduces the risk of some civilization diseases [10]. Carrots are a good source of carotenoids and dietary fiber and have beneficial health effects [11]. Drinking carrot juice enhances antioxidant activity and decreases lipid peroxidation and can reduce cardiovascular risk factors in adults. Beetroot juice contains high amounts of biologically active substances including betalains and inorganic nitrate [12]. Betalains are used as natural colorants in food production and they attract significant attention due to their possible health benefits in humans, especially their antioxidant and anti-inflammatory activities [13]. Tomatoes are a good source of vitamins and minerals, mostly ascorbic acid, α-tocopherol, folates and phenolic compounds. Tomato juice is rich in antioxidants, in particular lycopene, which has been shown to lower the risk of systemic inflammation, cardiovascular disease and prostate, lung, stomach and colorectal cancers [14].

Natural antioxidants play an important role in our diet, as they have been reported to possess beneficial bioactivities, including anti-allergic, antiviral, anti-inflammatory and anti-mutagenic properties [15]. Many studies are showing the effects of the addition of different vegetable parts such as pomace, dry product, oil, or seeds on baking goods [3,8]; however, the effect of juices has not been evaluated. Thus, to combine the increase of bread attractiveness and its nutritional value, our research focuses on different water substitution rates with carrot, tomato and beetroot juices, aiming to also benefit the sensory properties of the products.

## 2. Materials and Methods

### 2.1. Chemicals

Reagents and solvents used in this study were of the A.C.S. or HPLC purity and they were purchased from Merck (Darmstadt, Germany).

### 2.2. Research Material

The research material was the control wheat bread, bread with the addition of cloudy carrot, beetroot and tomato juices in three quantitative variants: 15%, 30%, and 50% in relation to the amount of water in the recipe. The bread was baked with the use of wheat flour type 750 produced by Polskie Młyny (Kluczbork, Poland) and pasteurized cloudy NFC (not from concentrate) vegetable juices produced by Szymanowice (Pleszew County, Poland). The characteristics of the added vegetable NFC juices are presented in Table 1.

### 2.3. Preparation of Bread

Breads in this experiment were baked applying the single-phase method on a laboratory scale applying the method described by Raczyk et al. [16]. The following ingredients were used: wheat flour type 750 from Teresin (Poland), tomato juice, beetroot juice, carrot juice, dried yeast, salt and water (Table 2). The breads were baked in two independent batches in a Sveba Dahlen oven (Fristad, Sweden) at 220 °C for 30 min. The obtained breads were stored in plastic containers in dark conditions at 25 °C and 60% RH until subsequent analysis.

### 2.4. Specific Volume of Bread

Each bread’s specific volume was measured and calculated according to the AACC Approved Method 10-05.01 [17] and expressed as mL/g.

### 2.5. Moisture and Ash Determination

Bread crumb and flour moisture, as well as ash content, were determined based on the AACC methods 44-15.02 and 08-01.01 [18,19] in the same way as described in our previous study [16].

### 2.6. Color Determination of Bread

The color of the bread crumbs were determined in five replications using a Konica Minolta CM-3600d colorimeter (Tokyo, Japan) set as we described in a previous publication [16]. The color parameters were measured in the CIE L*a*b* scale.

### 2.7. Evaluation of Breads Texture

The texture of the bread’s crumb samples was determined by texture profile analysis (TPA) in the same way as we described in our previous study [16,20]. Measurements were taken 24, 48, and 72 h after baking.

### 2.8. Active and Potential Acidity of the Bread Crumb

Ten g of each bread crumb lyophilizate (as a powder) was transferred to a 100 mL volumetric flask which was filled with distilled water. Ultrasonic extraction in a SW 3H device (SONO SWISS, Ramsen, Switzerland) was performed at room temperature for 10 min. The extracts were filtered through filter paper. Filtrates were used to determine the active acidity (pH) with the use of a HI 221 pH-meter coupled with a magnetic stirrer (Hanna Instruments, Poland). To determine the titratable acidity, 20 mL of the filtrate was taken. It was titrated with 0.1 M sodium hydroxide solution up to pH 8.1 with a titrator TitroLine^®^ 5000. The titratable acidity was expressed in degrees of acidity according to Sujka et al. [21].

### 2.9. Total Polyphenols Content

Total polyphenols content (TPC) was determined in samples according to Gao et al.’s [22] method with slight modifications. Briefly, 10 g of each bread (crumb with crust) lyophilizate (as a powder) was weighed with a precision of 0.01 g and transferred to a 100 mL volumetric flask and made up with methanol/distilled water (80/20 *v*/*v*). Ultrasonic extraction was performed in a SW 3H device (SONO SWISS) at room temperature for 10 min. The extracts were filtered through filter paper, and the filtrates were taken to determine total polyphenols. In sequence, 1 mL of the extract, 0.4 mL of the Folin Ciocalteu reagent, 3.2 mL of distilled water and 2 mL of 15% sodium carbonate were measured into test tubes and mixed thoroughly. At the same time, a blank test was prepared in which the addition of the extract was replaced with methanol/water solution. The tubes prepared in this way were stored for one hour in a dark place at room temperature. The absorbance of the samples was then measured on a Shimadzu UV-1650PC spectrophotometer at a wavelength of 765 nm. Additionally, a standard curve with gallic acid was made. The content of total polyphenolic compounds was expressed as milligrams of gallic acid equivalents per 100 g of fresh bread (µg of GAE/100 g fresh weight).

### 2.10. Determination of Total Antioxidant Activity as Scavenging Capacity

The scavenging capacity of the extract of free and bound phenolics found in bread was determined using the stable 2,2-diphenyl-1-picrylhydrazyl radical (DPPH) [23]. Briefly, 1 mL of extract was obtained according to Section 2.9 and was added to 3 mL of methanol and 1 mL of freshly prepared 0.33 M DPPH solution. The mixture was then kept at room temperature in a dark room for 10 min. The absorbance was read on a Shimadzu UV-1650PC spectrophotometer at a wavelength of 517 nm, exactly at the 10th minute after preparing the mixture. Additionally, a standard curve with Trolox was made. Taking into account dilutions and the molar mass of Trolox, the result was expressed as a micromole of Trolox per 100 g of fresh bread (µmol TR/100 g). Determinations were performed in triplicate.

The antioxidant activity of the samples was also determined using the radical monocation of 2,2′-azinobis-(3-ethylbenzothiazoline-6-sulfonic acid) (ABTS^•+^) according to Re et al. [24], with some modifications. Briefly, 24 h before the analyses, an ABTS^•+^ monocation radical solution was prepared, and it was ensured that the measured absorbance at 734 nm was in the range of 0.74–0.75. In the dark, 1 mL of extract was obtained according to Section 2.9, and was reacted with 4 mL of ABTS^•+^ solution for 10 min. The absorbance was then read on a Shimadzu UV-1650PC spectrophotometer at a wavelength of 734 nm. Additionally, a standard curve was made with Trolox. The ABTS value was expressed as a micromole of Trolox per 100 g of fresh sample (μmol TR/100 g). Measurements were performed in triplicate.

### 2.11. Sensory Evaluation of Breads

Ten trained panelists made sensory evaluations on the day of production 4 h after baking according to a previously published methodology [16]. A five-point scale was used for evaluation of overall acceptability, flavor, crust and crumb color, taste, and crumb porosity.

### 2.12. Calculations and Statistics

Statistica 13.3 (TIBCO Software Inc., Palo Alto, CA, USA) software was used to perform one-way ANOVA analysis with Tukey’s test. Principal component analysis (PCA) was used on the obtained data in order to visualize the changes in breads that have occurred as a result of replacement of the formula water with individual juices. The conditions for classifying data for PCA analysis are identical to those described earlier [25].

## 3. Results and Discussion

### 3.1. Characteristics of the Juices

Beetroot juice had the highest carbohydrates and total polyphenols content, therefore it was the most promising addition to breads among the juices used (Table 1). The measured TPC content of beetroot juice was about four times higher than the other juices. The lowest carbohydrate content was noticed in tomato juice. All of the juices were low (<1) in fat, protein and dietary fiber, so their addition to the breads did not contribute any content. Among the studied juices, carrot had the lowest titratable acidity, followed by beetroot, and tomato was the most abundant with organic acids (Table 1). With regard to pH, the lowest value was found in tomato juice. Similar values were obtained by other authors [3,9,11]. Possible differences in the characteristic values of each juice may be due to industry practices. To prolong the shelf life of carrot or beetroot juices, citric acid is normally added during processing to lower the pH [3,9]. Furthermore, the juices may have undergone different processing and thermal preservation under different parameters. The juices were completely different in color, but characteristic to the species, as evidenced by the measured color parameters L*, a*, and b*.

### 3.2. Specific Volume, Color and Texture of the Breads

**Figure 1:**
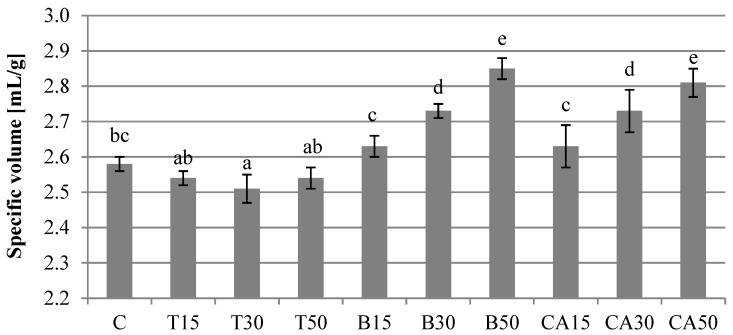
The specific volume of the analyzed breads. Bars represent means ± standard deviation, (*n* = 9, for each bread type). The values marked with different letters are significantly different (*p* < 0.05).

The volume of the loaves was significantly affected when beetroot and carrot juices substituted water in the bread-making process. An increase in the proportion of carrot and beetroot juices in the recipe (15, 30 and 50% *v*/*v*) resulted in a statistically significant increase in the loaves’ volume (Figure 1). Interestingly, tomato juice slightly decreased the volume of the breads. We suspect that the pH and carbohydrate content of the added juices played an important role in this case. The beetroot and carrot juices had the highest carbohydrate content and a pH of about 6.0, which improved the baking properties of the dough and increased the loaves’ volume.

**Figure A1:**
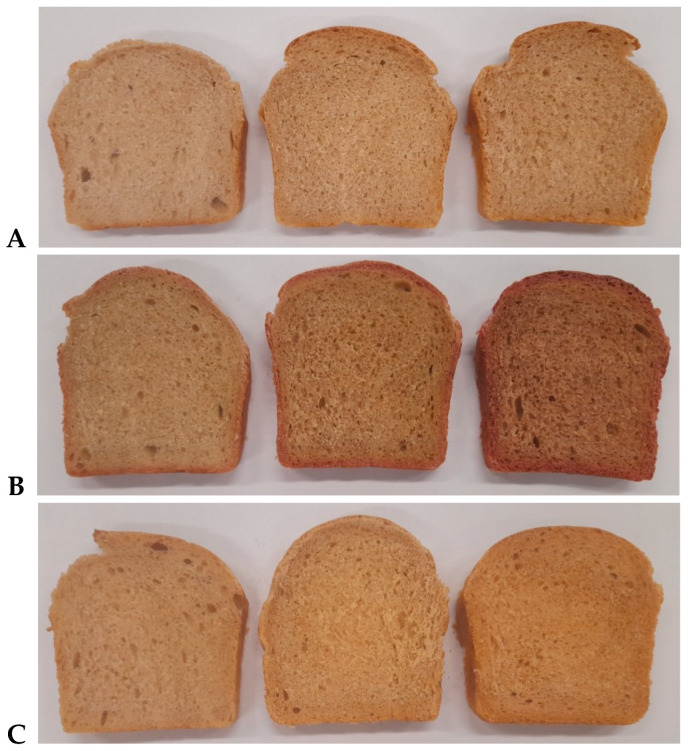
Pictures of the breads obtained in the experiment. (**A**)—breads with tomato juice; (**B**)—breads with beetroot juice; (**C**)—breads with carrot juice in horizontal order on each picture: breads with 15, 30, 50% of water replacement with juice.

The addition of juices also significantly affected the color of bread loaves. In greater proportion, regardless of the type of juice, the darker color the bread had (Table 3). Changes in other color parameters were also observed. The addition of juice significantly increased the redness of the bread (parameter a*). The yellowness (parameter b*) of each bread slightly increased in the case of tomato juice supplementation, and significantly in the case of the addition of other two juices, with the highest impact of carrot juice (Table 3, Appendix A Figure A1). This was influenced by the pigments present in the juices: carotenoids in carrot juice, especially β-carotene; red betacyanins and yellow vulgaxanthins in beetroot juice; and carotenoids in tomato juice, particularly lycopene. Other studies have also found an effect of natural dyes found in fruit pomace used to fortify breads [26,27]. Other authors also point to the influence of the Maillard reaction on the formation of crumb color, and even more on the crust [28,29]. However, the negligible protein content of the juices and the same baking conditions for all breads did not additively promote the Maillard reaction. Overall, the total color difference ΔE* calculated from the measured color parameters shows that even a 15% addition of juice relative to the amount of water in the recipe has markedly affected the color of wheat breads, which was noticeable to the human eye.

**Figure 2:**
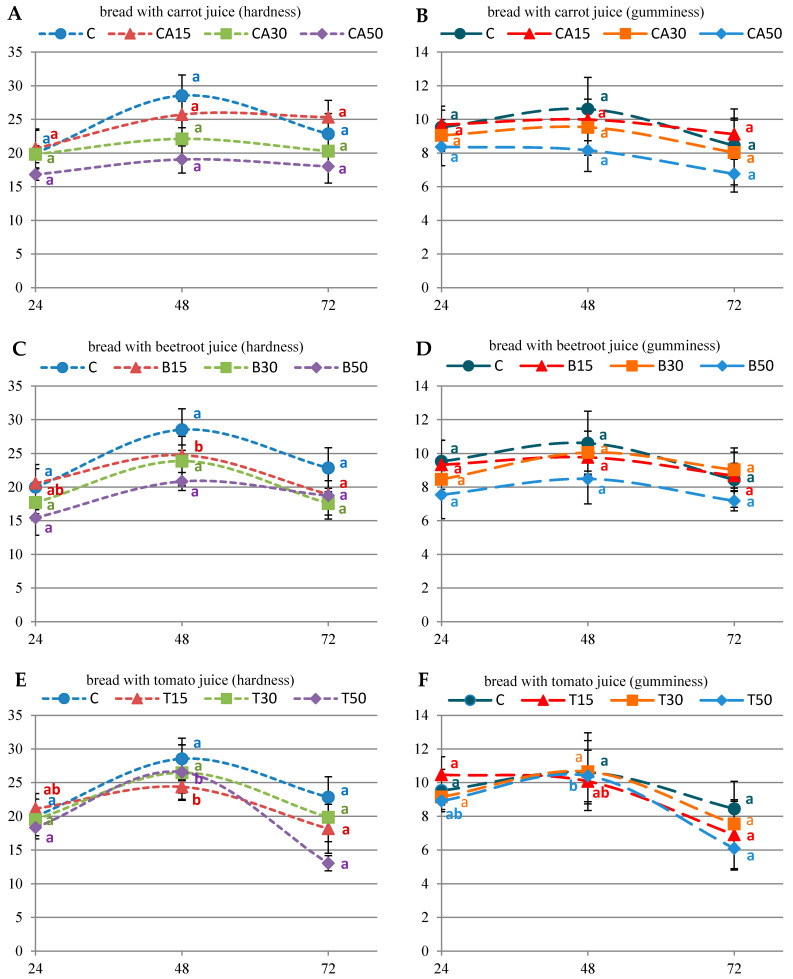
Crumb hardness [N] and gumminess during three days of storage for the experimental bread made with the formulation without (C) or with carrot (CA) juice ((**A**) hardness; (**B**) gumminess); beetroot (B) juice ((**C**) hardness; (**D**) gumminess); tomato (T) juice ((**E**) hardness; (**F**) gumminess). Points represent means ± standard deviation, (*n* = 6, for each bread type). The values within each type of bread marked with different letters are significantly different (*p* < 0.05).

In all tested variants, the hardness of the bread crumb increased 48 h after baking and then decreased after 72 h (Figure 2). The shapes of the hardness and gumminess curves had a similar course, hence it was noticed that the addition of vegetable juices did not significantly change the texture during storage. The addition of tomato juice did not have a significant effect on the initial hardness and gumminess of the bread. However, in the case of the 50% beetroot and carrot juice addition, the bread after 24 h of baking was softer and less gummy compared to the control sample (Figure 2).

### 3.3. Active and Potential Acidity of the Bread Crumb

The addition of vegetable juices had a statistically significant effect on the pH formation of the bread (Table 4). Supplementation of bread with tomato juice caused a significant reduction in the pH compared to the control sample. The opposite effect was observed when the breads were supplemented with beetroot or carrot juice. A 50% replacement of the water in the recipe with beetroot juice caused the pH to rise from 5.39 to 5.64. In the case of carrot juice, a significant change in pH was already determined with a 30% replacement of water in the recipe. It should be noted that regardless of the type of juice used, the 15% replacement did not affect the pH of the bread samples. The pH changes in the breads were dictated by the pH specific to the type of juice. Tomato juice had a pH of 4.3, which is lower than the pH of the control breads (average 5.48), while the beetroot and carrot juices had a pH of 6.0 and 6.2, respectively, which is higher than the pH of the control breads.

Supplementation with vegetable juices also caused dynamic changes in the titratable acidity (TA) of wheat breads. Regardless of the percentage of juice in the recipe, the acidity of the breads statistically significantly increased compared to the control. The increase in acidity was dependent on the number of organic acids contained in the added juices. Tomato and beetroot juices rich in organic acids (Table 1) [30,31] raised the acidity of the breads the most, especially in the case of 50% recipe water replacement. Carrot juice, the most deficient in organic acids among the three used in the experiment (Table 1), caused a smaller increase in the acidity of the bread, not exceeding the value of 4.0.

Studies of pH and titratable acidity show that with the appropriate addition of juice to the dough, these two parameters can be shaped in wheat bread. Consequently, this affects the organoleptic perception of the bread.

### 3.4. Total Polyphenols Content and Antioxidant Activity

It is well known that polyphenols are beneficial for health, due to their antioxidant properties, which act as free radical scavengers and reduce oxidative stress in the human body. Fruits, vegetables and juices pressed from them are particularly rich in polyphenols [32,33]. Therefore, it seems necessary to check the effect of the addition of juices on the polyphenol content of the bread matrix and the resulting antioxidant properties.

The addition of vegetable juices to wheat bread affected the content of phenolic compounds (Table 4). However, the change was not always statistically significant. For example, the addition of tomato or carrot juice minimally raised the TPC value. The greatest changes in TPC content were caused by the addition of beetroot juice, where already a 15% share caused a significant increase in phenolic compounds in the sample. As our study showed, the thermal baking process did not degrade all phenolic compounds added to the juice. The increase in TPC content was due to the addition of juice and the release of phenolic compounds from the matrix. Previous studies indicate that soluble phenols are released from fermented dough during bread baking, and insoluble phenols are generally preserved [34,35]. Other researchers reported that the greatest increase in TPC content is in the crust of bread, as the phenolic acids are also incorporated into Maillard reaction products during baking [36,37].

The antioxidant activity of bread supplemented with vegetable juices, measured by ABTS and DPPH assays, shows the positive effect of the baking process on this type of attribute. Antioxidant capacity increased in all bread variants tested compared to the control sample. The smallest effect of the addition of juice was observed in samples with carrot juice. A slightly better effect was obtained with tomato juice and a significant, several-fold increase in the antioxidant activity of the bread was found when beetroot juice was added. However, the degree of increase in antioxidant capacity was not the same when we compared the values measured by the ABTS and DPPH methods. The DPPH method was more sensitive to changes in juice shares in wheat bread. Another study seems to confirm our finding [34], but others indicate that the ABTS method is more sensitive in this matter [38]. Differences in sensitivity that are reflected in disparities in the results can be attributed to the solubility and diffusivity of radicals in solution. Another co-occurring factor that fits into the explanation is the different reactivity of bioactive compounds (such as flavonoids, phenolic acids) with DPPH and ABTS radicals. To check whether the increase in phenolic compounds by the addition of juices had a direct effect on the antioxidant activity of bread, a Pearson correlation was performed between TPC content and ABTS and DPPH measurement values. Correlations of 0.797 (TCP-ABTS) and 0.893 (TPC-DPPH) were obtained, indicating a significant but not exclusive effect of phenolic compounds on shaping the antioxidant capacity of wheat bread. Therefore, it is concluded that along with the juice, other biologically active compounds showing free radical scavenging abilities were also supplied, as well as several other substances with antioxidant potential which were formed during baking.

### 3.5. Sensory Characteristics of Bread

**Figure 3:**
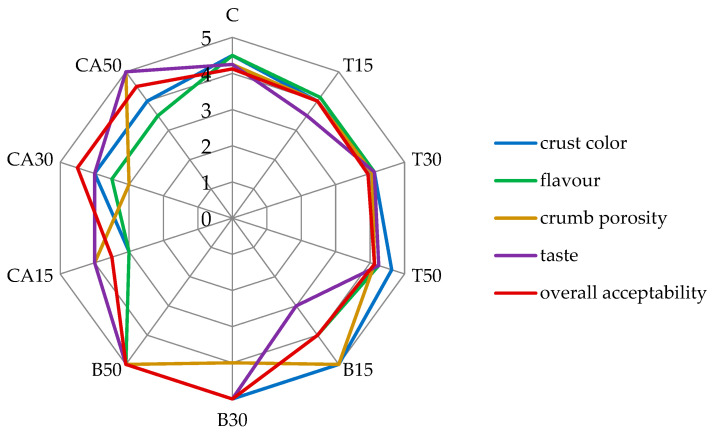
The scores of the sensory analysis of the obtained bread.

The addition of tomato juice did not significantly change the sensory characteristics of the bread according to the evaluation panel (Figure 3). However, carrot and beetroot juices had a significant impact on the sensory features. The most preferred and highest rated was the bread variant with 50% of beetroot juice. The 15% of carrot juice had a negative effect on the bread flavor, porosity and color, but its higher addition (50% replacement of water) had a positive effect on the taste and porosity of the crumb, but its flavor and crust color was rated lower than a control sample. Taking into account the overall acceptability of the bread with the addition of vegetable juices, the samples with 30% and 50% of beetroot and carrot juices obtained higher scores in the sensory evaluation compared to the control sample (Figure 3). A big influence on overall acceptability was the taste of the bread, shaped by acidity, flavor substances and aroma compounds contributed by the juices. Even though darker colors are undesirable in numerous foods, consumers believe that darker bread is healthier than lighter [39], so the addition of beetroot juice could positively influence this perception. Other research proved that the additives of vegetable purees contributed to the production of bakery products with high organoleptic characteristics and nutritional value [40].

### 3.6. The Comprehensive Overview of Obtained Bread Characteristics

**Figure 4:**
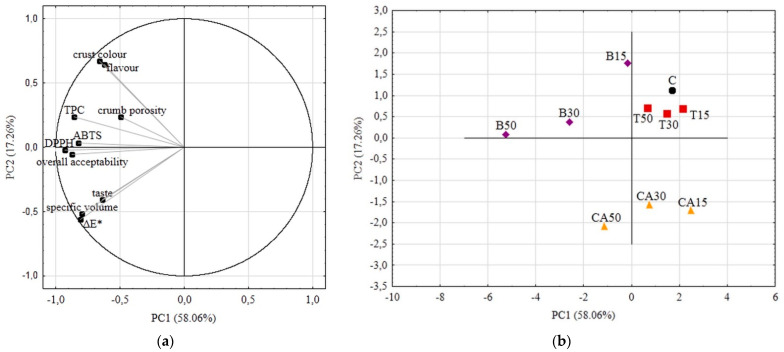
PCA analyses results. (**a**) Score plot, PC1 versus PC2 of all samples; (**b**) Score plot, PC1 versus PC2 of data from determinations used as variables.

A PCA chemometric analysis was conducted to evaluate and demonstrate changes in wheat breads, as influenced by the type and level of vegetable juice addition. The first two principal components explained 75.32% of the total variability. Figure 4a shows the distribution of samples in two-dimensional space relative to the first and second principal components.

The attributes that most differentiated the samples of bread with tomato juice were the total color difference (ΔE*), ABTS and DPPH values, and taste scores (Figure 4). As the proportion of tomato juice increased, the antioxidant activity of the bread increased. More red pigments caused marked changes in the color of the crumb, and increasing the content of organic acids changed the taste. However, not all PCA-qualified parameters differed as much as those mentioned above, so samples of tomato juice breads were grouped closely together, not far from the control sample. Therefore, we consider the addition of tomato juice to be inefficient in terms of changes in physicochemical, antioxidant and organoleptic properties.

Samples of wheat breads with beetroot or carrot juice additives were grouped into their respective subgroups, far from the control sample. In the case of breads with beetroot juice, the samples differed the most in antioxidant properties and TPC value, followed by organoleptic properties. The high content of bioactive compounds, which additionally act as colorants, caused significant positive changes in crust colour, crumb porosity, and flavour. Based on Figure 4b (distances between samples on the plane), it can be concluded that increasing the proportion of beetroot juice was the most efficient in terms of bread characteristics. Carrot juice bread samples varied the most in terms of taste, ΔE*, specific volume and antioxidant activity. The differences caused by the increasing levels of juice addition were not as distinct as in the case of beetroot juice, but were clearly separated from each other. We recognize that this juice is also suitable for enhancing the organoleptic and nutritional properties of wheat bread.

## 4. Conclusions

Based on the experiment results, it was concluded that carrot and beetroot juices contributed to more active fermentation resulted as increased bread volumes. The active and potential acidity of the bread crumbs was also significantly affected by the juices. By obtaining an intense color of bread with the addition of carrot and beetroot juice, it is possible to use them as natural food colorants in bakery products. Most importantly, the addition of vegetable juices proved to be a very good way to increase the antioxidant activity of wheat bread. PCA analyses revealed that carrot and beetroot juice are the most efficient in terms of shaping wheat bread properties. A 30% or 50% substitution of water for juice in a recipe increases the organoleptic value of baked goods, especially in terms of color and taste. The research showed that the addition of vegetable juices may be used in the bread production technology, creating a new assortment to meet consumers’ needs for food with high organoleptic and nutritional qualities.

## Figures and Tables

**Table 1:** Characteristics of vegetable juices used in the experiment.

**Measured Values**						
**Juice**	**TPC [mg GAE/100 mL]**	**pH**	**TA [g/L]**	L*	a*	b*
tomato	27.2 ± 1.3	4.3 ± 0.0	2.43 ± 0.05	35.1 ± 0.0	15.9 ± 0.1	11.6 ± 0.0
beetroot	115.7 ± 2.2	6.0 ± 0.0	2.03 ± 0.04	25.2 ± 0.04	3.7 ± 0.0	0.6 ± 0.0
carrot	30.0 ± 0.8	6.2 ± 0.0	1.83 ± 0.04	45.5 ± 0.1	28.0 ± 0.2	29.8 ± 0.2
**Nutritional Value per 100 g Taken from the Labels**						
**Juice**	**Energy** **[kJ/kcal]**	**Fat** **[g]**	**Carbohydrates [g]**	**Total Dietary Fiber** **(g)**	**Protein** **[g]**	**Salt** **[g]**
tomato	80/19	<0.5	4.0	0.4	<0.7	0.73
beetroot	162/38	0.1	8.7	0.3	0.2	0.188
carrot	135/32	0.1	6.0	0.4	0.7	0.073

TPC—total phenolic content; TA—titratable acidity; L*, a*, b*—colorimetric coefficients in CIELAB scale.

**Table 2:** Recipe ingredients of studied bread [% content].

Ingredients	C *	T15	T30	T50	B15	B30	B50	CA15	CA30	CA50
Wheat flour	61.4	61.4	61.4	61.4	61.4	61.4	61.4	61.4	61.4	61.4
Water	36.8	31.3	25.8	18.4	31.3	25.8	18.4	31.3	25.8	18.4
Juice	-	5.5	11.0	18.4	5.5	11.0	18.4	5.5	11.0	18.4
Salt	0.93	0.93	0.93	0.93	0.93	0.93	0.93	0.93	0.93	0.93
Yeast	0.87	0.87	0.87	0.87	0.87	0.87	0.87	0.87	0.87	0.87

* C—control (wheat bread), T, B, CA—bread with tomato, beetroot, or carrot juice addition, respectively; 15, 30, 50—percentage replacement of water with juice.

**Table 3:** Colorimetric coefficients in CIELAB scale of studied breads.

	L*	a*	b*	ΔE*
C	67.26 ± 0.37 de	3.49 ± 0.04 a	21.56 ± 0.13 a	-
T15	68.07 ± 1.13 e	4.94 ± 0.25 b	22.15 ± 0.32 b	2.05 ± 0.33 a
T30	66.51 ± 1.13 d	6.40 ± 0.25 c	22.91 ± 0.45 c	3.46 ± 0.40 b
T50	65.12 ± 0.76 c	7.98 ± 0.25 e	23.58 ± 0.35 d	5.40 ± 0.59 d
B15	64.57 ± 0.26 c	4.91 ± 0.11 b	24.68 ± 0.34 e	4.37 ± 0.30 c
B30	60.13 ± 0.78 b	8.07 ± 0.15 e	25.75 ± 0.24 f	9.47 ± 0.59 e
B50	54.06 ± 0.90 a	11.38 ± 0.32 g	24.37 ± 0.34 e	15.64 ± 0.84 g
CA15	66.71 ± 0.76 d	6.90 ± 0.28 d	26.03 ± 0.46 f	5.70 ± 0.53 d
CA30	67.70 ± 0.37 e	9.02 ± 0.26 f	29.18 ± 0.22 g	9.44 ± 0.30 e
CA50	65.45 ± 0.69 c	11.97 ± 0.19 h	32.65 ± 0.48 h	14.09 ± 0.54 f

Values represent means ± standard deviation (*n* = 6, for each bread type). The values in a column with different letters are significantly different (*p* < 0.05).

**Table 4:** Physicochemical and antioxidant properties of studied bread.

	Moisture (%)	Ash (%)	pH	TA (°Acidity)	TPC (µg GAE/100 g)	ABTS (µmol TR/100 g) *	DPPH (µmol TR/100 g) *
C	43.8 ± 0.2 a	1.09 ± 0.30 a	5.48 ± 0.05 b	3.17 ± 0.05 a	188 ± 2 a	249 ± 9 a	108 ± 20 a
T15	43.7 ± 0.4 a	1.20 ± 0.26 a	5.44 ± 0.02 b	3.71 ± 0.01 bc	195 ± 3 b	605 ± 12 c	208 ± 12 b
T30	43.0 ± 0.5 a	1.22 ± 0.14 a	5.37 ± 0.02 ab	3.88 ± 0.06 c	189 ± 0 a	674 ± 14 d	329 ± 16 c
T50	43.0 ± 0.6 a	1.29 ± 0.24 a	5.25 ± 0.03 a	4.39 ± 0.06 d	197 ± 5 b	744 ± 12 e	452 ± 16 d
B15	42.8 ± 0.3 a	1.19 ± 0.16 a	5.39 ± 0.03 b	3.85 ± 0.05 c	252 ± 1 e	1005 ± 16 f	373 ± 36 cd
B30	43.0 ± 0.5 a	1.24 ± 0.14 a	5.47 ± 0.03 b	3.93 ± 0.03 c	235 ± 2 d	1099 ± 7 g	585 ± 24 e
B50	42.6 ± 0.4 a	1.34 ± 0.21 a	5.64 ± 0.04 c	4.57 ± 0.05 d	318 ± 4 f	1126 ± 7 g	1383 ± 36 f
CA15	43.8 ± 0.1 a	1.16 ± 0.26 a	5.41 ± 0.04 b	3.52 ± 0.12 b	186 ± 1 a	531 ± 9 b	155 ± 16 ab
CA30	42.8 ± 0.3 a	1.19 ± 0.11 a	5.64 ± 0.09 c	3.96 ± 0.06 c	205 ± 0 c	568 ± 9 bc	323 ± 32 c
CA50	42.7 ± 0.4 a	1.34 ± 0.14 a	5.70 ± 0.02 c	3.93 ± 0.09 c	189 ± 4 a	669 ± 10 d	432 ± 12 d

* values expressed on 100 g of fresh weight; TA—titratable acidity; TPC—total phenolic content; Values represent means ± standard deviation (*n* = 3, for each bread type). The values in a column with different letters are significantly different (*p* < 0.05).

## Data Availability

All data created and analyzed during the experiments was presented in this study.

## Appendix A

**Table A1:** Crumb texture parameters for the selected analyzed bread after 24, 48 and 72 h of storage at room temperature.

	Resilience	Cohesiveness	Chewiness (N)	Resilience	Cohesiveness	Chewiness (N)
	C			T15		
**24 h**	0.88 ± 0.04 a	0.48 ± 0.03 b	8.33 ± 0.85 a	0.90 ± 0.08 a	0.50 ± 0.04 a	9.51 ± 1.59 ab
**48 h**	0.98 ± 0.02 b	0.37 ± 0.01 a	10.41 ± 1.63 a	0.97 ± 0.04 a	0.41 ± 0.01 a	9.81 ± 0.23 b
**72 h**	1.00 ± 0.03 b	0.37 ± 0.03 a	8.50 ± 1.87 a	0.93 ± 0.06 a	0.38 ± 0.09 a	6.35 ± 1.63 a
	T30			T50		
**24 h**	0.89 ± 0.05 a	0.47 ± 0.02 b	8.18 ± 0.05 a	0.89 ± 0.01 a	0.49 ± 0.02 a	7.97 ± 0.57 b
**48 h**	0.98 ± 0.03 a	0.40 ± 0.03 a	10.42 ± 2.26 a	0.94 ± 0.11 a	0.39 ± 0.00 a	9.71 ± 1.36 b
**72 h**	0.95 ± 0.04 a	0.38 ± 0.03 a	7.17 ± 1.56 a	0.83 ± 0.16 a	0.47 ± 0.12 a	4.95 ± 0.19 a
	B15			B30		
**24 h**	0.87 ± 0.07 ab	0.45 ± 0.03 a	8.09 ± 0.66 ab	0.83 ± 0.05 ab	0.48 ± 0.03 ab	7.05 ± 1.26 a
**48 h**	0.95 ± 0.04 b	0.39 ± 0.02 a	9.26 ± 0.65 b	0.98 ± 0.02 b	0.42 ± 0.02 a	9.89 ± 1.31 a
**72 h**	0.80 ± 0.01 a	0.46 ± 0.08 a	6.96 ± 0.50 a	0.78 ± 0.10 a	0.51 ± 0.03 b	7.15 ± 1.88 a
	B50			CA15		
**24 h**	0.87 ± 0.07 a	0.49 ± 0.01 b	6.57 ± 1.32 a	0.91 ± 0.06 a	0.47 ± 0.02 b	8.84 ± 1.42 a
**48 h**	0.97 ± 0.01 b	0.41 ± 0.01 a	8.26 ± 1.41 a	0.98 ± 0.04 a	0.39 ± 0.01 a	9.74 ± 0.75 a
**72 h**	0.96 ± 0.02 ab	0.39 ± 0.04 a	6.88 ± 0.74 a	0.99 ± 0.02 a	0.38 ± 0.03 a	11.02 ± 3.58 a
	CA30			CA50		
**24 h**	0.93 ± 0.08 a	0.46 ± 0.02 a	8.38 ± 0.73 a	0.92 ± 0.08 a	0.50 ± 0.04 b	7.63 ± 0.49 a
**48 h**	0.94 ± 0.09 a	0.43 ± 0.05 a	8.83 ± 0.82 a	0.95 ± 0.07 a	0.43 ± 0.02 ab	7.73 ± 0.78 a
**72 h**	0.92 ± 0.08 a	0.39 ± 0.05 a	7.26 ± 1.25 a	0.96 ± 0.07 a	0.38 ± 0.06 a	6.52 ± 1.19 a

Values represent means ± standard deviation (*n* = 6, for each bread type). For each bread, the values in a column with different letters are significantly different (*p* < 0.05).
